# ACOX2 is a prognostic marker and impedes the progression of hepatocellular carcinoma via PPARα pathway

**DOI:** 10.1038/s41419-020-03291-2

**Published:** 2021-01-04

**Authors:** Qifan Zhang, Yunbin Zhang, Shibo Sun, Kai Wang, Jianping Qian, Zhonglin Cui, Tao Tao, Jie Zhou

**Affiliations:** 1 Department of General Surgery, Division of Hepatobiliopancreatic Surgery, Nanfang Hospital, Southern Medical University, 1838 North of Guangzhou Avenue, Guangzhou, Guangdong 510515 China; 2 Shanghai Institute of Biochemistry and Cell Biology, Chinese Academy of Sciences, University of Chinese Academy of Sciences, Shanghai, 200031 China; 3 Department of Anesthesiology, Central People’s Hospital of Zhanjiang, 236 Yuanzhu Road, Zhanjiang, Guangdong 524045 China

**Keywords:** Cancer genomics, Mechanisms of disease, Diagnostic markers

## Abstract

Hepatocellular carcinoma (HCC) has been extensively studied as one of the most aggressive tumors worldwide. However, its mortality rate remains high due to ideal diagnosis and treatment strategies. Uncovering novel genes with prognostic significance would shed light on improving the HCC patient’s outcome. In our study, we applied data-independent acquisition (DIA) quantitative proteomics to investigate the expression landscape of 24 paired HCC patients. A total of 1029 differentially expressed proteins (DEPs) were screened. Then, we compared DEPs in our cohort with the differentially expressed genes (DEGs) in The Cancer Genome Atlas, and investigated their prognostic significance, and found 183 prognosis-related genes (PRGs). By conducting protein–protein interaction topological analysis, we identified four subnetworks with prognostic significance. Acyl-CoA oxidase 2 (*ACOX2*) is a novel gene in subnetwork1, encodes a peroxisomal enzyme, and its function in HCC was investigated in vivo and in vitro. The lower expression of ACOX2 was validated by real-time quantitative PCR, immunohistochemistry, and Western blot. Cell Counting Kit-8 assay, wound healing, and transwell migration assay were applied to evaluate the impact of ACOX2 overexpression on the proliferation and migration abilities in two liver cancer cell lines. ACOX2 overexpression, using a subcutaneous xenograft tumor model, indicated a tumor suppressor role in HCC. To uncover the underlying mechanism, gene set enrichment analysis was conducted, and peroxisome proliferator-activated receptor-α (PPARα) was proposed to be a potential target. In conclusion, we demonstrated a PRG ACOX2, and its overexpression reduced the proliferation and metastasis of liver cancer in vitro and in vivo through PPARα pathway.

## Introduction

Hepatocellular carcinoma (HCC) is the third leading cause of cancer death in the world^1–3^. Chronic inflammation infection, mainly caused by hepatitis B virus (HBV) or hepatitis C virus (HCV), has been proposed as a causative mechanism in HCC carcinogenesis^4^. Although the diagnosis and treatment methods of HCC have been developing, exairesis is still the first line and most effective treatment to HCC^5^. What is frustrating is that the recurrence and mortality of HCC is still high because of the extensive characteristics of HCC, including intrahepatic metastasis, venous invasion, and distant metastasis^6^. Therefore, exploring new targets for early diagnosis and treatment of HCC is a common goal.

Proteomics is a powerful tool that accurately monitors and quantitatively detects changes in protein expression^7^, and has been widely used to identify and quantitatively detect HCC-related proteins^8–10^. Data-independent acquisition (DIA) is an emerging technology for quantitative proteomic analysis of large samples^11^. It combines deep proteome coverage capabilities with quantitative consistency and accuracy^12^. DIA proteomic technology has been widely used in the diagnosis and research of diseases, such as breast cancer^13^, glioblastoma^14^, prostate cancer^15,16^, kidney cancer^17^, and ovarian cancer^18^. Acyl-CoA oxidase 2 (*ACOX2*) is a member of the ACOX protein family, which maps to chromosome 3p14.3^19^. ACOX2 encodes branched-chain acyl-CoA oxidase, a peroxisomal enzyme believed to be involved in the metabolism of branched-chain fatty acids and bile acid intermediates^20^. ACOX2 deficiency has been reported in several liver-related diseases, such as persistent hypertransaminasemia^21^. Also, it has been reported that ACOX2 was related to carcinogenesis in human colorectal cancer^22^. Zhou and Wang^23^ found that ACOX2 was deficient in primary malignant cardiac tumors. Bjorklund et al.^24^ reported that ACOX2 could promote the proliferation of estrogen receptor-positive (ER+) breast cancers. However, the roles of ACOX2 in liver cancer have not been well characterized.

In this study, we conducted a DIA proteome analysis on HCC tissue (CA) and adjacent nontumorous tissue (NO) samples. We found that the ACOX2 protein level was decreased in HCC tissue compared to that in adjacent nontumorous tissue. Low expression of ACOX2 predicts a poor prognosis. Moreover, we demonstrated that ACOX2 could suppress liver cancer proliferation and migration in vitro and in vivo.

## Materials and methods

### Patients and tissue samples

A total of 24 pairs of HCCs and the corresponding noncancerous tissue specimens used for proteomics examination were simultaneously obtained from 24 HCC patients in Nanfang Hospital, Southern Medical University. Another 30 pairs of HCCs and adjacent nontumor samples were used for validation. All clinical samples in vitro were stored at −80 °C. Patients were not subjected to any neoadjuvant therapy before surgery. The patients’ information was obtained from medical charts and follow-up. Informed consent was obtained from each patient. The study was approved by the Protection of Human Subjects Committee of Nanfang Hospital (approval no. NFEC-201801-K4). The clinicopathological data of the patient’s samples in the proteomics analysis are summarized in Supplementary Table S1.

### Protein extraction and trypsin digestion

All the samples were minced and lysed in 8 M urea-containing protease and phosphatase inhibitors (Thermo Fisher Scientific, MA, USA). The lysate was centrifuged at 14,000 × *g* for 10 min and the supernatant was collected. Protein concentration was determined by the Bradford protein assay. One hundred micrograms of proteins per sample was dissolved in 100 μL of 8 M urea. Two microliters of 0.5 M TCEP (tris(2-carboxyethyl)phosphine) was added and the sample was incubated at 37 °C for 1 h, then 4 μL of 1 M iodoacetamide was added to the sample, and the incubation lasted for 30 min. Prechilled acetone was added to precipitate the proteins overnight at −20 °C. The precipitates were washed with 1 mL of prechilled 90% acetone aqueous solution twice and then redissolved in 100 μL of 100 mM TEAB (tetraethylammonium tetrahydroborate). Sequence-grade-modified 1:50 (enzyme:protein, weight:weight) trypsin (Promega, Madison, WI) was added to digest the proteins at 37 °C. The peptide mixture was desalted by C18 ZipTip, and quantified by Pierce quantitative colorimetric peptide assay (Thermo Scientific, MA, USA).

### Spectral library generation

For the spectral library, the peptide mixture of the 48 samples redissolved in 20 mM ammonium formate, and then fractionated by Ultimate 3000 system (Thermo Fisher Scientific, MA, USA) connected to a reverse-phase column (XBridge C18 column, Waters Corporation, MA, USA). High pH separation was performed by a linear gradient, starting from 5 to 45% 20 mM ammonium formate in 40 min. The column was re-equilibrated at the initial condition for 15 min. Ten fractions were collected and each fraction was dried in a vacuum concentrator. The fractions were redissolved in 0.1% formic acid and analyzed by nanospray liquid chromatography with tandem mass spectrometry (LC-MS/MS) on an Orbitrap Fusion Lumos Tribrid (Thermo Fisher Scientific, MA, USA) coupled to Waters nanoACQUITY UPLC System (Waters, MA, USA). Two microliters of peptide was loaded to the analytical column (Acclaim PepMap C18, 75 μm × 25 cm) and separated with 120-min gradient, from 3 to 30% of 0.1% formic acid. The mass spectrometer was run under DDA mode, and automatically switched between MS and MS/MS mode. The DDA data were processed and analyzed by Spectronaut X (Biognosys, Schlieren, Switzerland) with default settings to generate an initial target list. Spectronaut X was set up to search the UniProt database of *Homo sapiens* (downloaded on 21 May 2019). Carbamidomethyl (C) was specified as the fixed modification, and oxidation (M) as the variable modification. False discovery rate (FDR) cutoff on precursor and protein level was applied at 1%.

### Protein identification and quantitation

DIA was performed with 45 isolation windows, each window overlapped 1*m*/*z*, the total cycle time was 3.98 s. The DIA data were processed and analyzed by Spectronaut X (Biognosys, Schlieren, Switzerland) with default settings. Retention time prediction type was set to dynamic iRT. Spectronaut X determined the data extraction and the ideal extraction window dynamically, depending on iRT calibration and gradient stability. FDR cutoff on precursor and protein level was <1%. Decoy generation was set to apply a random number of amino acid position swamps (min = 2, max = length/2). Otherwise, all the selected fragment ions passing the filters were used for quantification. The MS proteomics data have been deposited to the ProteomeXchange Consortium (http://proteomecentral.proteomexchange.org) via the iProX^25^ partner repository with the dataset identifier PXD021979.

### Multivariate data analysis

The multivariate data matrix was analyzed by the EZinfo software 2.0 (Waters Corp., Milford, MA, USA) to cope with highly multivariate, noisy, collinear, and possibly incomplete data. Orthogonal partial least squares discrimination analysis (OPLS-DA) was used to establish the relationship between protein expression and samples, and then various samples could be separated.

### DEP identification and functional analysis

To identify DEPs between HCC and adjacent nontumorous tissues, *P* value <0.05 and |fold change (FC)| > 2 were selected as the criteria for DEPs. In order to obtain an overview of the characteristics of DEPs, heatmap with hierarchical clustering analysis was performed based on the normalized values of all proteins using R package. Gene enrichment analysis was performed based on KEGG, gene ontology, and *P* < 0.05 was set as the cutoff for significantly enriched functional GO terms or KEGG pathways. Protein–protein interaction was performed by the String database and visualized by Cytoscape (version 3.4.0).

### Gene set enrichment analysis

Gene set enrichment analysis (GSEA) was used to screen the significantly changed pathways^26^. Preranked GSEA was performed with 1000 permutations. The *P* value was calculated by family-wise error rate, which is a robust method for multiple testing^27^. The GSEA plots were visualized by limma R package^28^.

### Cell lines and cell culture

Human hepatoma cell lines (HepG2, SMCC-7721, Hep3B, and MHCC97H), the normal human hepatocyte cell line (HL-7702), were brought from Cell Bank of Chinese Academy of Sciences (Shanghai, China). The cells were cultured in high-glucose Dulbecco’s modified Eagle’s medium with 10% fetal bovine serum and 1% antibiotics, and incubated in a humidified atmosphere at 37 °C with 5% CO_2_ and 95% air.

### Cell transfection

The full-length messenger RNA (mRNA) of ACOX2 was cloned into the Lenti-CMV vector, and the lentivirus was produced as previous study^29^. The lentivirus exhibiting ACOX2 overexpression was transfected into HepG2 and SMCC-7721 cells, and the positively transduced cells were selected using puromycin.

### Cell counting kit-8 (CCK-8) assay

Cellular viability was determined by CCK-8 (Beyotime Biotechnology). Briefly, 100 μL of HepG2 or SMCC-7721 cells per well were plated into 96-well plates. After the corresponding reagent treatment, 10 μL of CCK-8 solution was incubated in the cell medium for 2 h at 37 °C. The absorbance of each well was detected at 450 nm by Multiskan FC Microplate spectrophotometer.

### Transwell migration assay

Human hepatoma cells were cultured with 10 μg/mL mitomycin C (Biochempartner, Shanghai, China) for 3 h. Then, cells (2 × 10^5^/well) were plated into the top chamber and 10% FBS-containing medium was placed into the bottom chamber. After incubation at 37 °C in 5% CO_2_ for 24 h, the cells remaining at the upper surface of the membrane were removed with a cotton swab. Meanwhile, the invaded or migrated cells, which adhered to the lower surface, were stained with 0.1% crystal violet and measured by optical microscopy.

### Wound-healing assay

ACOX2 overexpression or vector-transduced HepG2 and SMCC-7721 cells (5 × 10^5^ cells/well) were seeded into 6-well plates (Corning) with 10 μg/mL mitomycin C (Biochempartner, Shanghai, China) for 3 h. Wounds were made by scratching the adherent cells on the plate with a sterile 200-μL pipette tip (12 h after seeding), replaced with fresh culture medium, and then cultured for 24 h. The migration ability was evaluated by analyzing the migration of the cells into the wounded area.

### Real-time quantitative reverse transcription PCR (RT-qPCR)

Total RNA was extracted using TRIzol reagent (Life Technologies) according to the manufacturer’s instructions. RT-qPCR was performed using the SYBR Green qPCR Master Mix (Applied Biosystems) according to the manufacturer’s instructions. glyceraldehyde 3-phosphate dehydrogenase (GAPDH) was used as the internal control. The sequences of specific primers used in this study are listed below:

ACOX2-F: 5′-AGCACCCCGACATAGAGAGC-3′ and ACOX2-R: 5′-CTGCGGAGTGCAGTGTTCT-3′; GAPDH-F: 5′-GGAGCGAGATCCCTCCAAAAT-3′ and GAPDH-R: 5′-GGCTGTTGTCATACTTCTCATGG-3′; PPARA-F: 5′-ATGGTGGACACGGAAAGCC-3′ and PPARA-R: 5′-CGATGGATTGCGAAATCTCTTGG-3′; PPARD-F: 5′-CAGGGCTGACTGCAAACGA-3′ and PPARD-R: 5′-CTGCCACAATGTCTCGATGTC-3′; PPARG-F: 5′- GGGATCAGCTCCGTGGATCT-3′ and PPARG-R: 5′- TGCACTTTGGTACTCTTGAAGTT-3′.

### Western blot

Total protein was extracted from RIPA lysis buffer (Invitrogen) and the concentration was determined using BCA Protein Assay Kit (Beyotime, Shanghai, China). The protein samples were separated by 10% sodium dodecyl sulfate-polyacrylamide gel electrophoresis gels and then transferred onto polyvinylidene difluoride membranes. After blocking for 1 h at room temperature, the membranes were probed with primary antibodies overnight at 4 °C. Then, the membranes were incubated for 1 h in the specific horseradish peroxidase-conjugated secondary antibodies at room temperature. All images were developed by the ECL Kit (Servicebio, Wuhan, China) and obtained by using Bio-Rad ChemiDoc XRS+ Imaging System (USA). Antibodies against ACOX2 (#PA5-18670), PPARA (#PA1-822A), and PPARD (#PA1-823A) were purchased from Invitrogen (Thermo Fisher, USA), and PPARG (#ab178860) and GAPDH (#ab8245) were provided by Abcam (UK). GAPDH was used as an internal control for protein quantities.

### Tumorigenesis assays in nude mice

Thirty male BALB/c-nu/nu mice (Shanghai SLAC Laboratory Animal Co., Ltd.), aged 6 weeks, were randomly divided into three groups and were subcutaneously injected with 1 × 10^7^ ACOX2 or vector-transfected HepG2 cells. The tumor volume was assessed 18 days after injection. All the mice were euthanized on day 33, and the tumor weight was evaluated. Animal experiments and all experimental protocols were approved by the Ethics Committee of Nanfang Hospital, Southern Medical University. All experiments were performed in accordance with national and institutional guidelines and regulations.

### In vivo metastasis assay

HepG2 cells (control, vector-ov, ACOX2-ov, 1 × 10^6^ in 200 µL of phosphate-buffered saline) were injected into the tail vein of male BALB/c nude mice (*n* = 6/group). All mice were scanned using the IVIS@Lumina III system after 36 days. Then, the mice were killed painlessly. The lung tissues were isolated, pictured, fixed in formalin, and prepared into paraffin-embedded sections. Hematoxylin and eosin (HE) staining was performed using the HE Staining Kit (Vector Labs, Burlingame, CA, USA) and lung tissues were counted for metastatic nodules under a light microscope. At least five random sections per lung tissue were analyzed.

### Statistical analysis

GraphPad Prism 7.0 was used for the statistical analyses. Data are presented as the mean ± SD. Statistical significance was determined using Student’s *t* test. A *P* value <0.05 indicated the statistical significance.

## Results

### Proteomic profiling and DEPs in HCC

**Fig. 1:**
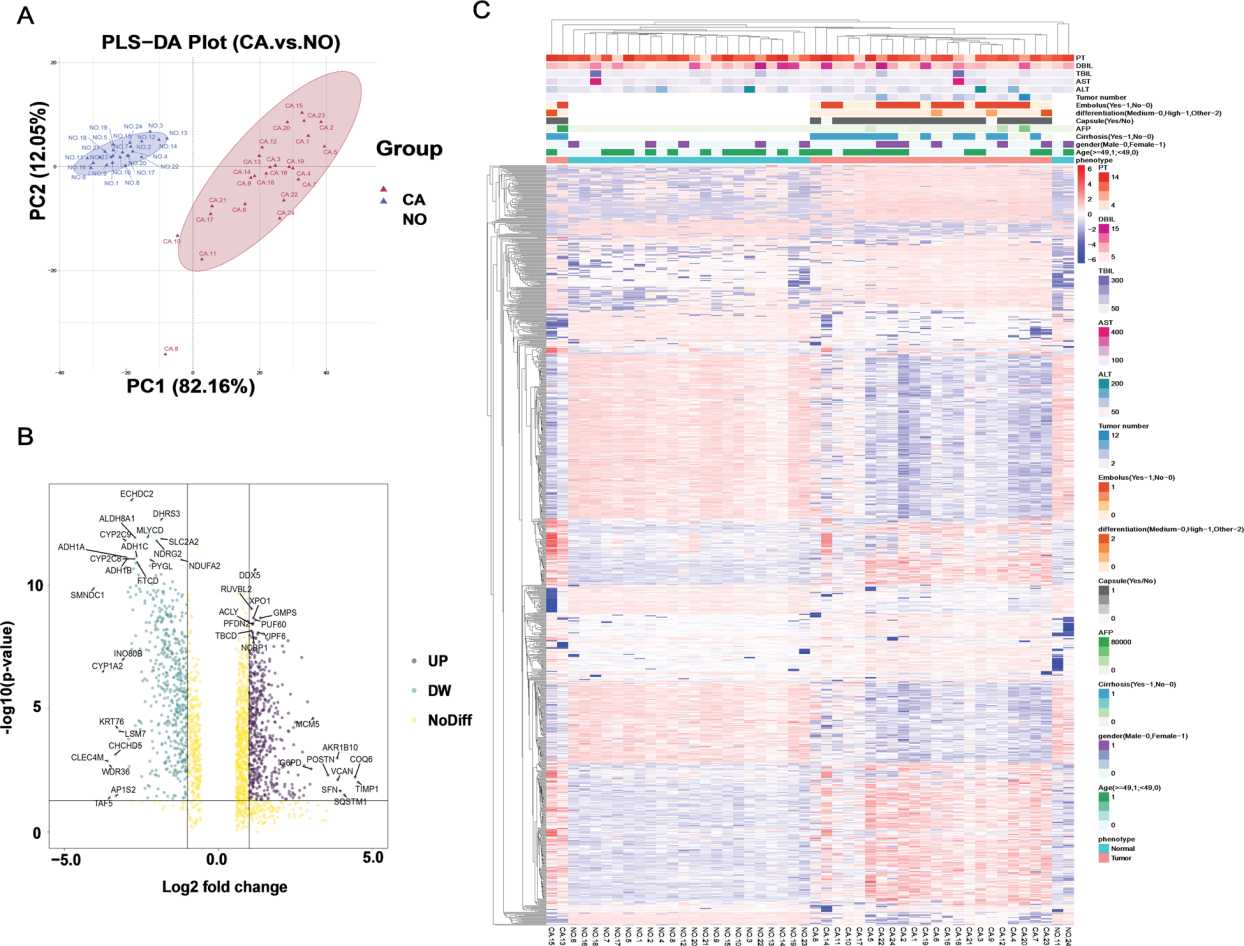
The DEP identification and correlation analysis with HCC clinicopathological traits. **A** OPLS-DA analysis of protein expression in hepatocellular carcinoma (CA) and paired adjacent normal (NO) samples. **B** Volcano plot showing significantly differentially expressed proteins. UP upregulated proteins were labeled in purple, DW downregulated proteins were labeled in green, NoDiff proteins with no significant differential expression. **C** Hierarchical clustering of differentially expressed proteins in hepatocellular carcinoma (CA) and paired adjacent normal (NO) samples. Clinical traits, including PT, DBIL, TBIL, AST, ALT, tumor number, embolus, differentiation, capsule, AFP, cirrhosis, gender, and age, were shown.

In this study, 24 pairs of samples, including coupled HCC and adjacent nontumorous tissues, were selected and subjected to DIA proteome analysis. As a result, 5459 proteins were identified and 1029 proteins were significantly differentially expressed in carcinomas compared with adjacent nontumor tissues (DEPs, |FC| > 2, *P* < 0.05), including four reiterated proteins. All of the DEPs were summarized in Supplementary Table S2. To study and visualize the discrepancy between CA and NO, OPLS-DA was applied to assess the data of the proteome of 24 HCC tissues and 24 adjacent nontumorous tissues, by which an obvious separation of CA and NO proteomic profiling was shown and suggested the significant difference between protein patterns of the two groups (Fig. 1A). Next, a volcano plot was applied to show the DEPs selected with the criterion described above and DEP names with the largest FC and/or −log 10 (*P* value) were labeled (Fig. 1B). As expected, mass genes were located at the center of the *x*-axis, indicating no change between CA and NO profiling. On the contrary, 554 proteins located to the left were upregulated in CA groups and 475 proteins located to the right were downregulated in CA groups. Furthermore, hierarchical clustering analysis was applied to classify the 1029 DEPs described above. As a result, 22 NO and 22 CA samples were separated clearly, and eight clusters were identified in total and labeled in different colors. Otherwise, some important clinical information of 24 patients, including PT, DBIL, TBIL, AST, ALT, tumor number, embolus, differentiation, capsule, AFP, cirrhosis, gender, and age, was labeled (Fig. 1C).

### Functional enrichment analysis of common differentially expressed genes (DEGs)

**Fig. 2:**
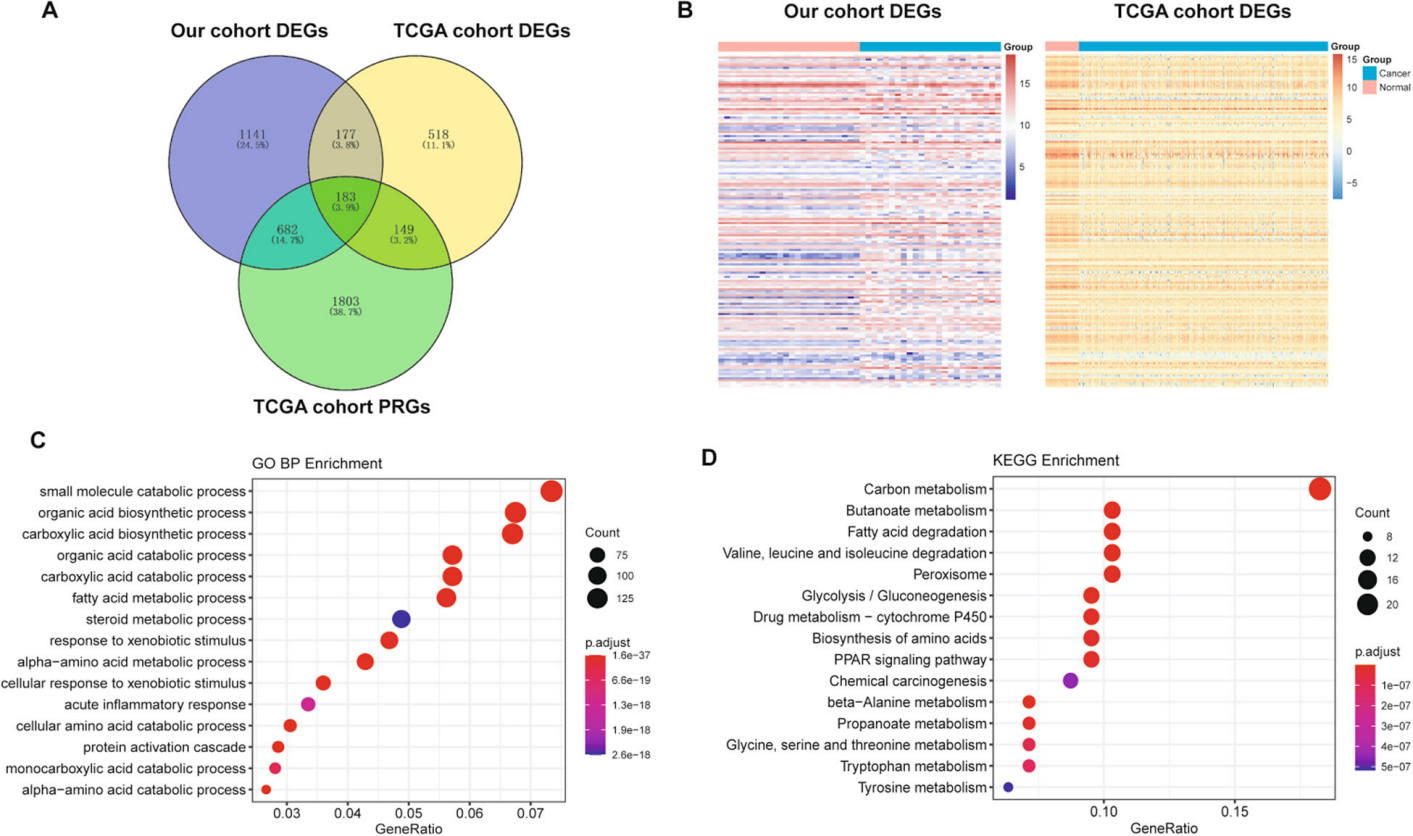
Functional enrichment analysis of common DEGs. **A** The common genes between our DEGs and TCGA cohort DEGs, PRGs of HCC. **B** The heatmap of the 161 common DEGs in our cohort and TCGA cohort of HCC. The red color indicates the normal samples and blue color indicates the HCC samples. **C** The top 15 significantly enriched GO-biological processes of the 161 DEGs **D** The top 15 significantly enriched KEGG pathways of the 161 common DEGs.

We first compared our DEPs, The Cancer Genome Atlas (TCGA) cohort DEGs, and TCGA cohort prognosis-related genes (PRGs) of HCC, and found 183 common genes (Fig. 2A). Among these genes, 161 DEG expression trends were consistent in the protein and mRNA levels (Fig. 2B). Next, we performed the GO-biological process (Fig. 2C) and KEGG pathway enrichment analyses (Fig. 2D). As a result, some important pathways are related to liver function or cancer genesis and development, such as fatty acid metabolism and some amino acid metabolism.

### PPI network construction and subnetwork identification

**Fig. 3:**
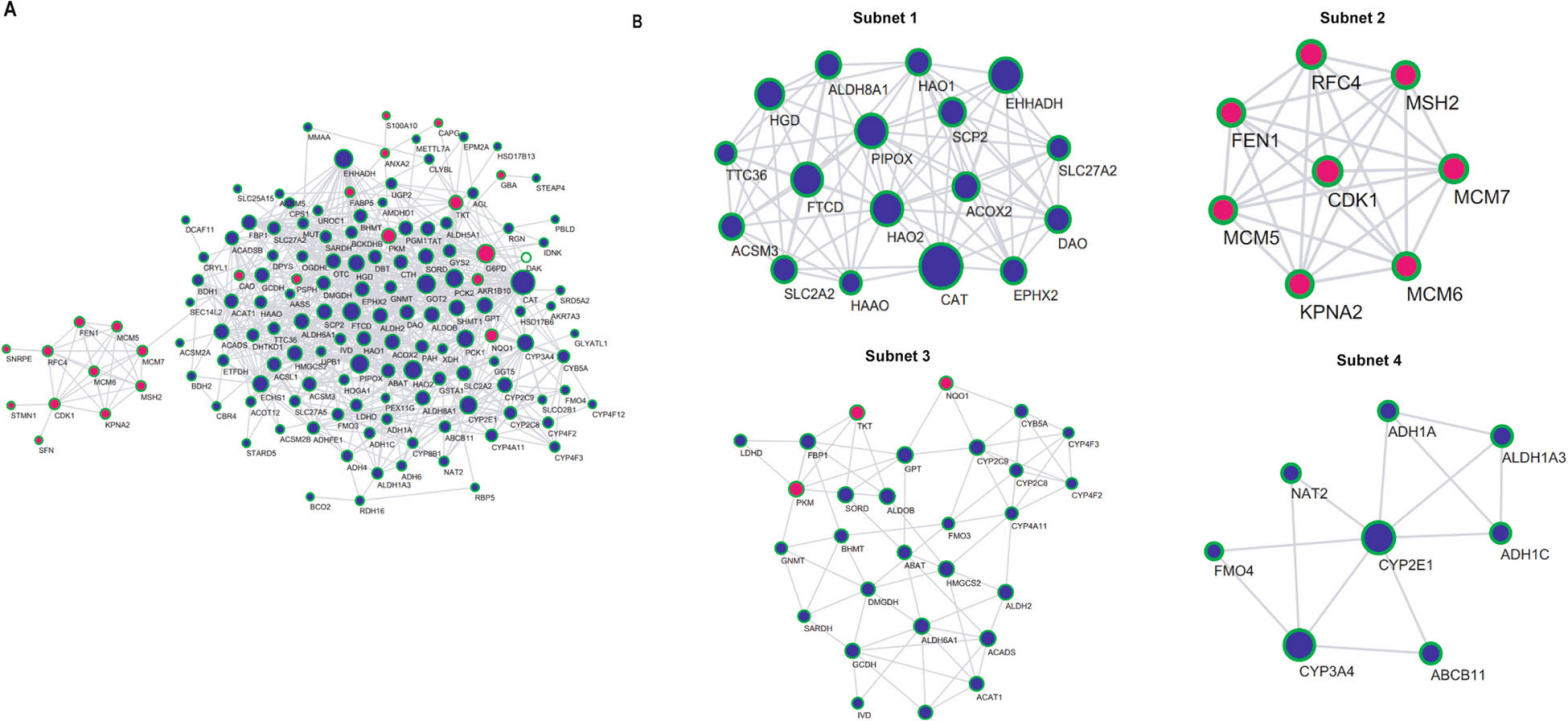
Protein–protein interaction network of 161 common DEGs. **A** Protein–protein interaction network of 161 common DEGs. Upregulated DEGs were labeled in red and downregulated DEGs were labeled in blue; *P* value and fold change were log transformed, and applied as the bolder with and node fill color; topological degree was used as the criteria for node size. **B** Four key subnetworks from the main network. The subnetworks were based on the results of the score calculation using the MCODE plugin of Cytoscape.

**Fig. 4:**
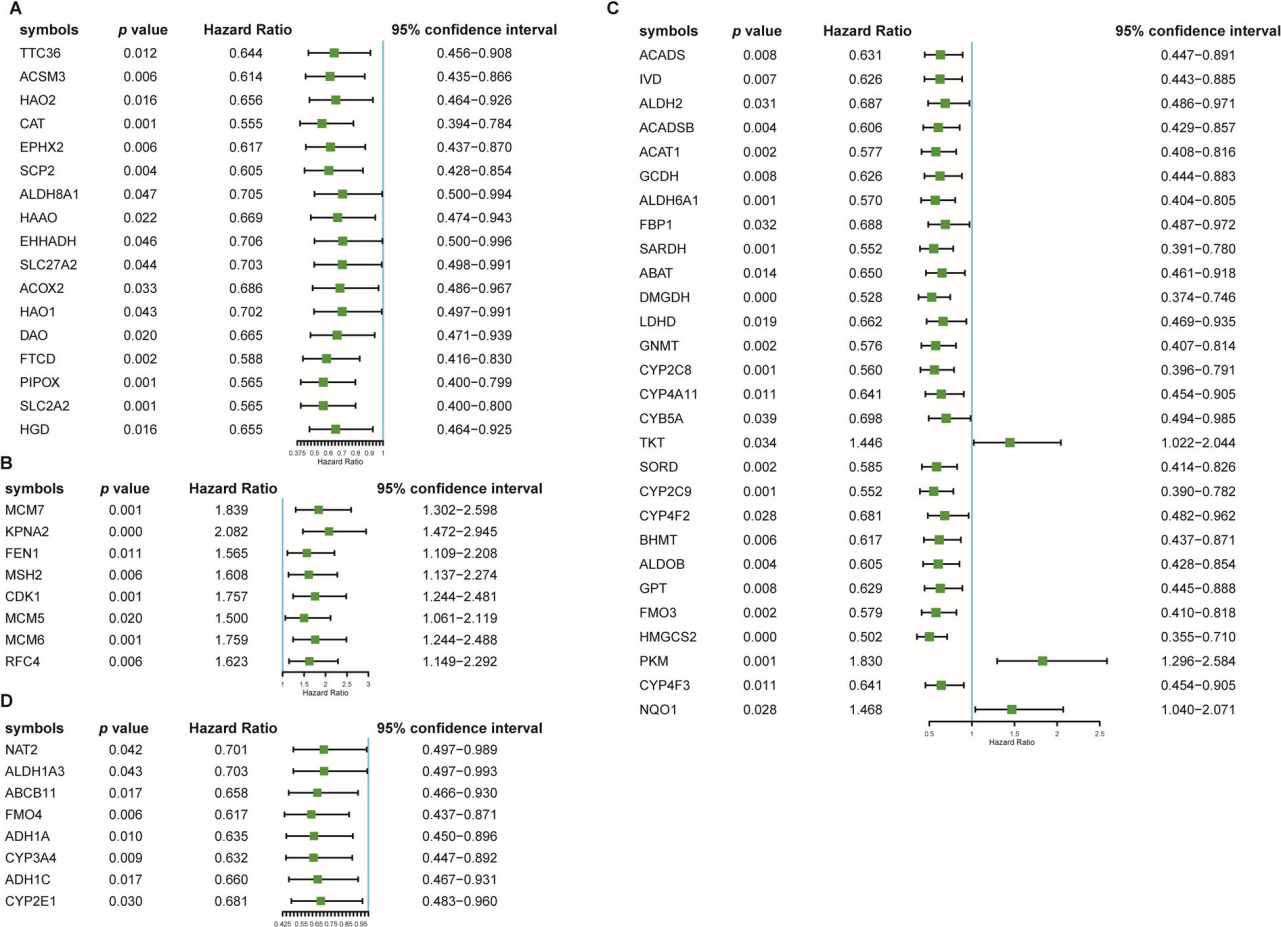
Forest plot of the four subnetworks. **A** Forest plot of the genes in subnet 1. **B** Forest plot of the genes in subnet 2. **C** Forest plot of the genes in subnet 3. **D** Forest plot of the genes in subnet 4. The forest plot showed the *P* value, hazard ratio (HR), and 95% CI of the DEGs.

To systematically analyze the biological functions of the 161 DEGs, a PPI network was constructed based on the STRING database and was visualized by Cytoscape 3.4.0 (Fig. 3A). The network contains 142 nodes and 715 edges. It is well acknowledged that subnetwork analysis of genes plays an important role in integrated biological networks. Based on the results of the score calculation using the MCODE plugin of Cytoscape, the most four significant modules were identified to have relatively high scores in the regulatory network (Fig. 3B). Then, the forest plot showed the *P* value, hazard ratio (HR), and 95% confidence interval of the DEGs in the four networks (Fig. 4). We found all the genes’ HR < 1 in subnets 1 and 4, HR of most genes in subnet 2 and subnet 3 also <1, which means these genes were not risk factors in hepatocarcinogenesis.

### ACOX2 is downregulated in liver cancer

**Fig. 5:**
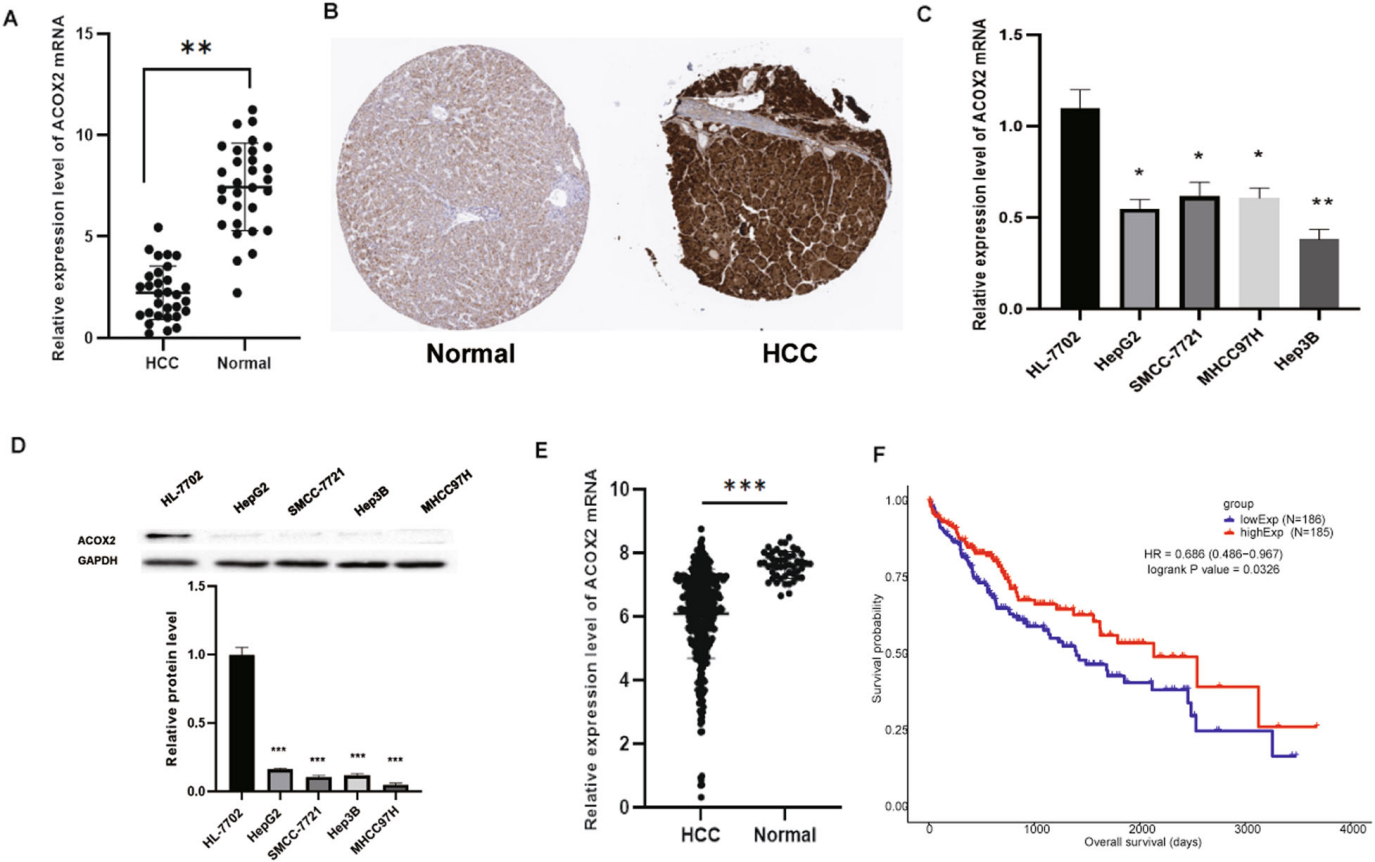
ACOX2 is downregulated in liver cancer. **A** The expression of *ACOX2* gene in liver cancer tissues and paired paracancer tissues by using RT-qPCR. **B** The expression of ACOX2 protein in liver cancer tissues and normal control tissues was detected by using immunohistochemistry. The expression distribution of ACOX2 in normal liver tissue and HCC patient samples was evaluated in the THPA database. **C** The expression of ACOX2 mRNA in normal liver cells and liver cancer cells by using RT-qPCR (*P < 0.05 and **P < 0.01). **D** The protein expression of ACOX2 in normal liver cells and liver cancer cells by Western blot (***P < 0.001). **E** The mRNA expression of ACOX2 in the TCGA dataset (***P < 0.001). **F** Survival analysis of the association between ACOX2 and overall survival time in HCC patients (based on the TCGA data, *P* = 0.0326).

Among the 161 genes above, we did a search in PubMed (https://pubmed.ncbi.nlm.nih.gov/). We found that three genes *ACOX2*, *ALDH8A1*, and *SCP2* were not reported in-depth in liver cancer. Then, statistical analysis was performed based on gene expression level and clinical information (Supplementary Table S3). We finally found that only ACOX2 was significantly correlated with cirrhosis (*P* < 0.05). So, ACOX2 was selected for further study. We next verified the expression of ACOX2 in liver cancer tissues. RT-qPCR was performed to detect ACOX2 expression in 30 pairs with HCC and the adjacent noncancerous tissues. The results showed that the expression of ACOX2 in liver cancer tissues was much lower than that from the adjacent noncancerous tissues (Fig. 5A). Immunohistochemistry experiment from the THPA database^30,31^ (https://www.proteinatlas.org/) was used to examine ACOX2 expression in liver cancer tissues and adjacent noncancerous tissues. We found that the positive rate of ACOX2 was much lower in tumor tissues than control tissues (Fig. 5B). Further, we detected the expression of ACOX2 in liver cancer cells and normal human hepatocyte cells. We found that the ACOX2 mRNA level was much lower in HepG2 and SMCC-7721 cells than in HL-7702 cells (Fig. 5C). Finally, we validated our results by using the TCGA data, the result is consistent with our research (Fig. 5D). Survival analysis showed that low expression reduced the overall survival probabilities of liver cancer patients (*P* = 0.0326) (Fig. 5E).

### Inhibition of cell growth and migration of HCC cells by ACOX2 in vitro and in vivo

**Fig. 6:**
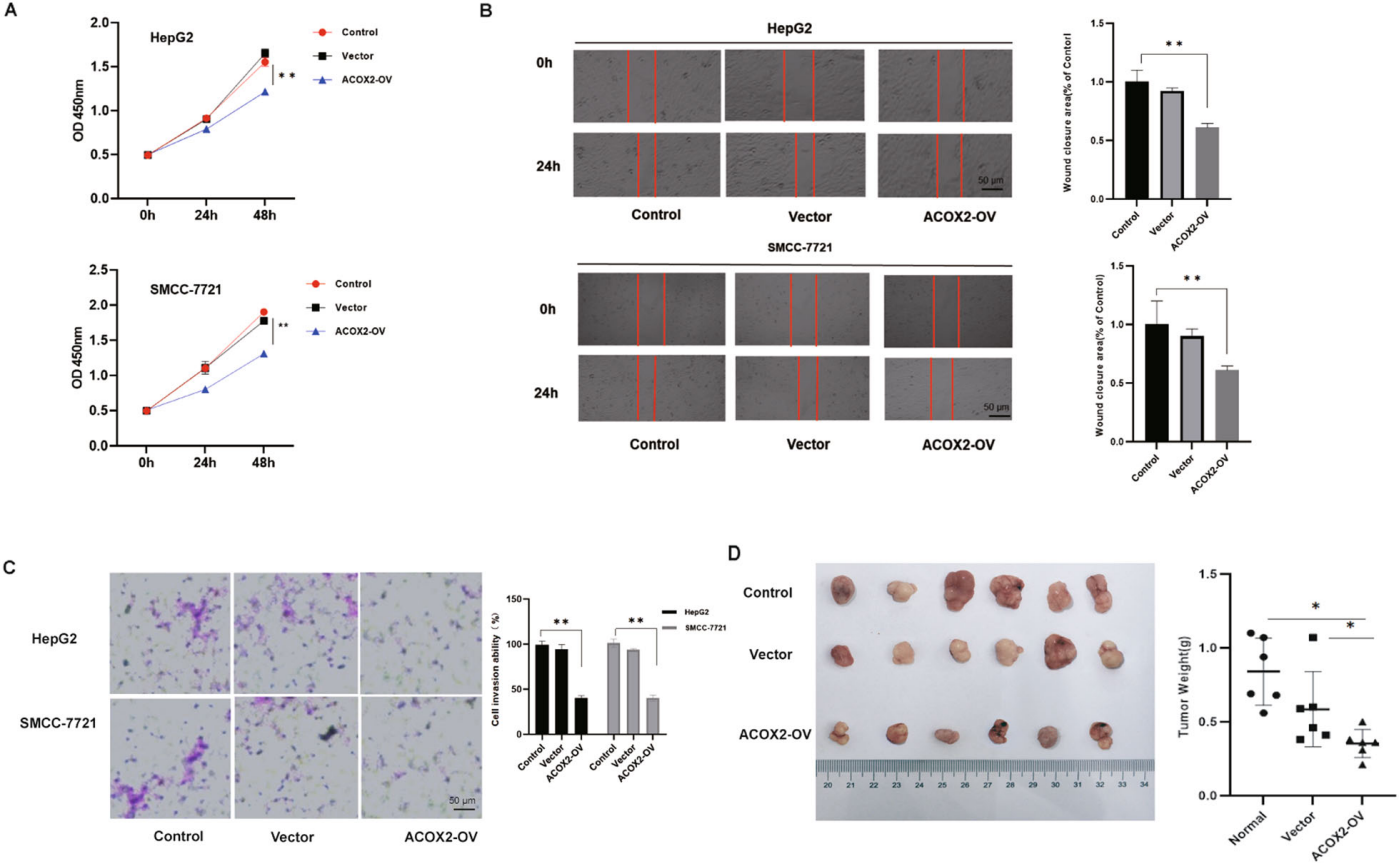
ACOX2 overexpression inhibits liver cancer cell proliferation and migration. **A** ACOX2 overexpression inhibits HepG2 and SMCC-7721 cells growth by CCK-8. **B** ACOX2 overexpression inhibits migration of HepG2 and SMCC-7721 cells. The wound-healing ability was determined 24 h after scratching. **C** ACOX2 overexpression inhibits invasion of HepG2 and SMCC-7721 cells. The effects of ACOX2 on liver cancer cell invasion were determined by transwell assays. **D** ACOX2 inhibits liver cancer cells in vivo. ACOX2 inhibits tumor growth of HepG2 cells in nude mice. The control, ACOX2-overexpressing, or vector-infected HepG2 cells were injected into nude mice (*n* = 6). Tumor weight was displayed above. *P < 0.05 and **P < 0.01.

**Fig. 7:**
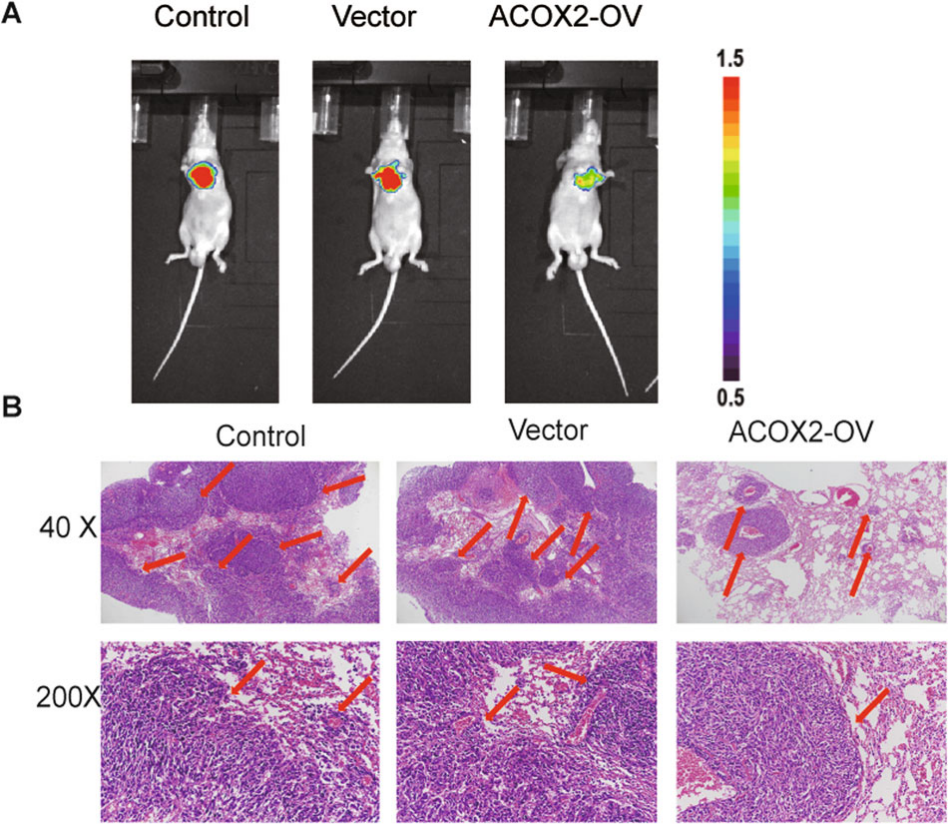
ACOX2 overexpression inhibits tumor lung metastasis. **A** Representative in vivo imaging of control, ACOX2-overexpressing, or vector-infected HepG2 cells in nude mice after 36 days. **B** Representative HE staining images of lung metastatic nodules (indicated by red arrow).

To determine the function of ACOX2 in liver cancer, we first overexpressed ACOX2 in HepG2 and SMCC-7721 cells and found that overexpression of the ACOX2 inhibits the proliferation of the HepG2 and SMCC-7721 cells (Fig. 6A). Then, we performed the wound-healing experiments on the liver cancer cells with control, vector, and ACOX2 overexpression and found that the migration ability was significantly decreased upon ACOX2 overexpression (Fig. 6B). Furthermore, transwell migration assays were performed to determine the effect of ACOX2 on migration. As shown in Fig. 6C, the migrated cells were significantly reduced with the overexpression of ACOX2. Together, these experiments demonstrated that ACOX2 induces cell growth inhibition and reduced migration ability of HepG2 and SMCC-7721 cells. To further examine the function of ACOX2 in vivo, we applied a xenograft murine model and injected the HepG2 cells into the nude mice in the blank group, vector group, and ACOX2 overexpression group. Consistent with the in vitro results, cells with ACOX2 overexpression inhibited the growth of the HepG2 cells. The tumor weight was significantly lower in cells transduced with ACOX2 overexpression plasmid (Fig. 6D). Consistently, ACOX2 overexpression decreased tumor growth and lung metastasis in xenograft tumor (Fig. 7A, B). Together, these results revealed an antitumor activity of ACOX2 in vitro and in vivo.

### ACOX2 inhibits tumor through the peroxisome proliferator-activated receptor-α (PPARα) signaling pathway

**Fig. 8:**
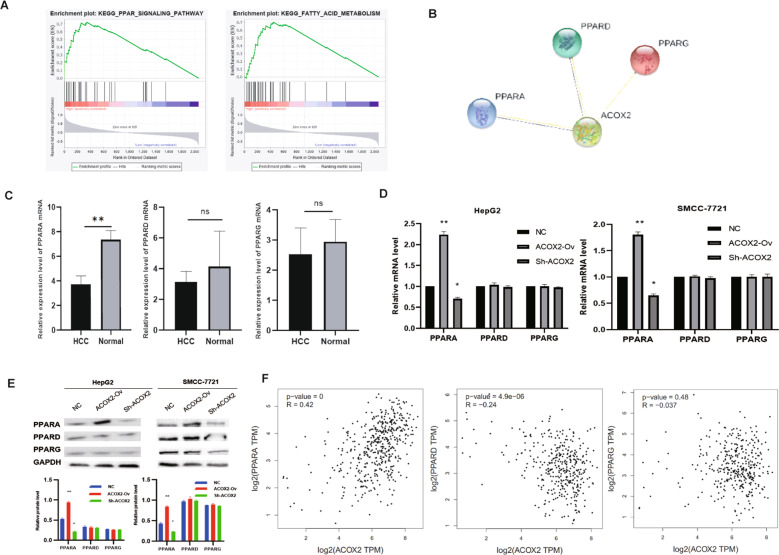
ACOX2 inhibits tumor through the PPARα signaling pathway. **A** The GSEA showed that PPAR (*P* = 0.004) and FATTY_ACID_METABOLISM (*P* = 0.006) were significantly changed. **B** The PPI network of ACOX2, PPARA, PPARD, and PPARG was constructed by the String database. **C** The expression of PPARA, PPARD, and PPARG genes in liver cancer tissues and adjacent noncancerou tissues by using RT-qPCR. **D** The mRNA expression of PPARA, PPARD, and PPARG was detected by RT-qPCR in HepG2 and SMCC-7721 (NC, the normal HCC cell lines; ACOX2-Ov, overexpression of ACOX2; Sh- ACOX2, knockdown ACOX2). **E** The protein levels of PPARA, PPARD, and PPARG were detected by Western blot in HepG2 and SMCC-7721 (NC, the normal HCC cell lines; ACOX2-Ov, overexpression of ACOX2; Sh- ACOX2, knockdown ACOX2, *P < 0.05; **P < 0.01; n.s. not significant). **F** The Pearson correlation analysis between ACOX2 and PPARA, PPARD, and PPARG by the GEPIA database.

To investigate the direct pathway that was regulated by ACOX2, a GSEA analysis was performed by low and high ACOX2 expression in our liver cancer proteomics data. Following this analysis, we found that several pathways like PPAR_SIGNALING_PATHWAY (*P* = 0.004) and FATTY_ACID_METABOLISM (*P* = 0.006) were significantly enriched in cells with ACOX2 high expression (Fig. 8A). By using the String database, we found that ACOX2 was associated with PPARA, PPARD, and PPARG that belong to the PPAR pathway (Fig. 8B). To explore the mechanism of ACOX2 in suppressing liver cancer proliferation, expression levels of PPARA, PPARD, and PPARG were validated by RT-qPCR using the previous tumor samples (Fig. 8C). Also, the RT-qPCR and Western blot of HepG2 and SMCC-7721 cells with ACOX2 overexpression and knockdown were performed. We found that only PPARA expression was consistent with ACOX2 (Fig. 8D, E). The results clearly demonstrate that low expression of ACOX2 in cancer cells significantly inhibits the expression of PPARA, but not PPARD and PPARG. Further, the expression of PPARA is positively correlated with ACOX2 in liver cancer (Fig. 8F). So, ACOX2 may regulate the PPARα pathway to inhibit the proliferation of liver cancer.

## Discussion

The incidence of HCC is the highest in several developing countries and it is progressively increasing in the developed world^32^. As the metastasis and multidrug resistance of HCC, the 5-year survival rate of patients with HCC is in the range of only 5%^33^. In addition, due to the aggressive behavior of HCC, the difficulties to reach an early diagnosis, and the modest effectiveness of curative treatments, the number of deaths worldwide per year is comparable to the incidence number^34^. Over the past decade, the “omics”-based methods have been widely used in identifying valuable novel targets for HCC^35,36^.

In the present study, we compared the proteomic profile of HCC tumor tissue with matched nontumorous tissue by a DIA LC-MS-based quantification strategy. A total of 1029 DEPs were identified. Combining analysis with the PRGs in TCGA, we found 161 common genes. The altered expression of these genes may act as messengers that transport signals between cells to promote liver cancer progression.

ACOX2 is the rate-limiting enzyme in the β-oxidation of branched, long-chain fatty acids and in the synthesis of bile acid precursor molecules^37^. At present, there are few studies about the function of ACOX2 in cancers. A previous study showed that ACOX2 may be as a potential novel therapeutic biomarker in ER+ breast tumors^24^. D’Arcy et al.^38^ found that lower expression of ACOX2 showed good prognosis among luminal A breast tumors of African-American (AA) women. In our study, we found that ACOX2 was significantly downregulated in liver cancer tissues and cells. Also, ACOX2 inhibited the proliferation and metastasis of hepatocellular carcinoma cells.

Through the GSEA analysis, we found that the PPARα pathway was activated in tumor samples with higher ACOX2 expression. Also, PPARα and ACOX2 are strongly correlated in the liver cancer tissues. As a nuclear receptor, PPARα activation generally inhibits tumorigenesis through its antiangiogenic and anti-inflammatory effects^39^. You et al.^40^ found that PPARα reduced Glut1 protein and mRNA levels in several cancer cell lines, including HCT-116, SW480, HeLa, and MCF-7, which led to decreased influx of glucose in cancer cells. Chen et al.^41^ reported that fenofibrate, a common drug used to treat severe hypertriglyceridemia and mixed dyslipidemia, altered glucose and lipid metabolism, inhibited gastric cancer cell proliferation, and promoted apoptotic gastric cancer cells through PPARα. In colon cancer, intestinal PPARα protects against colon carcinogenesis via regulation of methyltransferases DNMT1 and PRMT6^42^. All these results showed that PPARα pathway plays a positive role in inhibiting tumor, which is consistent with our findings. However, a study about pancreatic cancer showed that PPARα produced significantly higher expression in pancreatic cancer tissues than in tumor-adjacent tissues, and the PPARα expression level is inversely associated with higher overall patient survival rate^43^. The absence of PPARα expression suppresses tumor growth of Lewis lung carcinoma cells^44^. Together, these two seemingly contradictory observations imply that the effect of PPARα may be two-pronged.

Taken together, our study deeply investigated HCC biology by a DIA LC-MS-based quantification strategy, which might shed light on understanding the tumorigenesis and development of HCC. However, more studies are needed to clarify the molecular mechanism responsible for the roles of ACOX2 in liver cancer.

## Supplementary information

TableS1 Patients characteristics

TableS2 Differently expressed proteins

TableS3 Clinical information
